# The Relationship Between Gut Microbiota and Inflammatory Diseases: The Role of Macrophages

**DOI:** 10.3389/fmicb.2020.01065

**Published:** 2020-06-09

**Authors:** Ji Wang, Wei-Dong Chen, Yan-Dong Wang

**Affiliations:** ^1^State Key Laboratory of Chemical Resource Engineering, College of Life Science and Technology, Beijing University of Chemical Technology, Beijing, China; ^2^Key Laboratory of Molecular Pathology, School of Basic Medical Science, Inner Mongolia Medical University, Hohhot, China; ^3^Key Laboratory of Receptors-Mediated Gene Regulation and Drug Discovery, Hebi People’s Hospital, School of Medicine, Henan University, Kaifeng, China

**Keywords:** gut microbiota, inflammatory diseases, macrophage, obesity, inflammatory bowel disease

## Abstract

Gut microbiota, an integral part of the human body, comprise bacteria, fungi, archaea, and protozoa. There is consensus that the disruption of the gut microbiota (termed “gut dysbiosis”) is influenced by host genetics, diet, antibiotics, and inflammation, and it is closely linked to the pathogenesis of inflammatory diseases, such as obesity and inflammatory bowel disease (IBD). Macrophages are the key players in the maintenance of tissue homeostasis by eliminating invading pathogens and exhibit extreme plasticity of their phenotypes, such as M1 or M2, which have been demonstrated to exert pro- and anti-inflammatory functions. Microbiota-derived metabolites, short-chain fatty acids (SCFAs) and Gram-negative bacterial lipopolysaccharides (LPS), exert anti-inflammatory or pro-inflammatory effects by acting on macrophages. Understanding the role of macrophages in gut microbiota-inflammation interactions might provide us a novel method for preventing and treating inflammatory diseases. In this review, we summarize the recent research on the relationship between gut microbiota and inflammation and discuss the important role of macrophages in this context.

## Introduction

Gut microbes, an essential part of the microbiota ecosystem, outnumbers human cells by 10-fold (Zhang et al., 2015). The gut microbiota contains bacteria, fungi, archaea, and protozoa and can be altered by the host genetics, overuse of antibiotics, and changes in diet, as described in the accompanying review (Belkaid and Hand, 2014; Pickard et al., 2017). The most abundant phyla in human intestine were *Firmicutes*, *Bacteroidetes*, *Proteobacteria*, and *Actinobacteria* (Tap et al., 2009). *Firmicutes* are gram-positive bacteria including the large class of *Clostridia* and the lactic acid bacteria. Lactic acid bacteria are marketed as probiotic which are benefit for human health. Another sort of gram-positive bacteria are *Actinobacteria*, which include *Collinsella* and *Bifidobacterium* spp. *Bifidobacteria* is the other probiotic, which has been made in functional foods. Conversely, *Bacteroidetes*, and *Proteobacteria* are gram-negative bacteria, and LPS on their surface can induce activation of macrophages toward pro-inflammatory phenotype. Both can cause infection or diseases under certain conditions. The gut microbiota plays such a critical role in human health and disease that it has been called the “forgotten organ” (O’Hara and Shanahan, 2006). During millions of years of coevolution, the gut microbiota has been living in a symbiotic relationship with the host and affecting the energy balance (Backhed et al., 2004). In addition, symbiotic bacteria promote the intestinal immune system maturity (Mazmanian et al., 2005) and protect against pathogen colonization (Kaiser et al., 2012). Changes in the gut microbial composition result in chronic inflammation and metabolic dysfunction, as has been reviewed elsewhere (Sommer and Backhed, 2013). It is worth noting that the microbiota metabolites, short-chain fatty acids (SCFAs), play a key role in colonic inflammation (Zeng et al., 2019). Many studies have shown that not only epithelial cells or neutrophils but also monocytes and macrophages are modulated by SCFAs (Correa-Oliveira et al., 2016).

Inflammation is a normal physiological response of the body to the foreign pathogen invasion and plays two conflicting roles in human health (Xie et al., 2013). On the one hand, inflammation is the body’s automatic defense response, which also promotes wound healing. On the other hand, excessive inflammatory response results in a series of diseases such as obesity, atherosclerosis, and cancer, which has been reviewed in elsewhere (Wellen and Hotamisligil, 2005; Galkina and Ley, 2009; Crusz and Balkwill, 2015). During acute inflammation, neutrophils are recruited to the inflamed tissue sites, while during chronic inflammation, lymphocytes, macrophages, and plasma cells accumulate and infiltrate the junction tissue (Hakansson and Molin, 2011). There is growing awareness that many prevalent diseases are linked to chronic inflammation. Thus, it is important to regulate inflammation in a timely manner to control the morbidity from disease (Tracey, 2002).

Macrophages are regarded as critical effectors of inflammation. Resident tissue macrophages perform specific functions in response to their local environment (Hume et al., 2002). For example, macrophages are Kupffer cells in the liver and microglia in the central nervous system (CNS). Historically, blood monocytes exit the blood, enter tissues and undergo terminal differentiation to become tissue-resident macrophages (Geissmann et al., 2010). More recently, evidence has shown that tissue-resident macrophages, including lung macrophages and Kupffer cells, are established before birth and complemented by recruited monocytes under inflammatory conditions (Yona et al., 2013). They express pattern recognition molecules, such as Toll-like Receptor (TLR) 4, to recognize foreign pathogens, remove foreign molecules, and protect against infection (Gordon, 2002). In addition, they respond to the inflammatory stimuli and differentiate into classically (“M1”) or alternatively (“M2”) activated macrophages. As reviewed by Hakansson and Molin (2011) macrophages infiltrate tissues during inflammation and perform major functions, including antigen presentation, phagocytosis, and production of various cytokines and growth factors to participate in immune regulation. It is worth mentioning that macrophages are pro-inflammatory under the Lipopolysaccharides (LPS) stimulation (Fujihara et al., 2003).

In this review, we summarize the current understanding of the link between gut microbiota and inflammation focusing on the roles of macrophages. In particular, we discuss two major inflammatory diseases, obesity and inflammatory bowel disease (IBD), and provide a description of the macrophages as the immune and inflammatory effector cells.

## Gut Microbiota and Inflammation

The gut microbiota and its metabolites may regulate the host inflammatory conditions (Yang et al., 2017; Feng et al., 2018). Numerous studies have linked the gut microbiota to inflammatory diseases. Forbes et al. (2018) have demonstrated that the immune-mediated inflammatory diseases, such as Crohn’s disease (CD), ulcerative colitis (UC), multiple sclerosis (MS), and rheumatoid arthritis (RA), change the composition of the gut microbiota. In addition, there are abundant reports highlighting the gut microbiota role in the pathogenesis of the inflammatory diseases such as asthma, type 1 and type 2 diabetes mellitus (DM), and obesity (Arrieta et al., 2015; Knip and Siljander, 2016; Meijnikman et al., 2018).

The 16S ribosomal RNA (rRNA) sequencing paved the way for comparing the gut microbiota composition among individuals, which revealed the correlation between specific microbes and disease (Supplementary Table S1). *Enterobacteriaceae*, a large class of gram-negative facultative bacteria, are commonly linked to many inflammation diseases like IBD and obesity (Zeng et al., 2017). For example, adherent-invasive *E. coli* (AIEC) has been associated with CD, while diffusely adherent *E. coli* (DAEC) has been linked to UC (Mirsepasi-Lauridsen et al., 2019). In addition, the depletion of the phyla *Firmicutes* and the increase of the *Proteobacteria* populations have been linked to human IBD (Matsuoka and Kanai, 2015). Moreover, an increase in the ratio of *Firmicutes* to *Bacteroidetes* populations has been associated with obesity (Turnbaugh et al., 2006). Zhang et al. (2013) reported that the high levels of the phylum *Firmicutes* and the class *Clostridia* are found in diabetic patients. Zhu et al. (2013) found that children with non-alcoholic steatohepatitis (NASH) had a significantly higher proportion of *Proteobacteria*. Additionally, the gut microbiota of the RA patients contained higher levels of *Lactobacillus* and *Prevotella copri* and, correspondingly, the numbers of *Bifidobacteria* and *Bacteroides* decreased (Liu et al., 2013; Scher et al., 2013; Supplementary Table S1).

Short-chain fatty acids the major metabolic products of the gut microbiota digestion of the non-absorbable dietary fiber and resistant starches, included acetate, propionate, and butyrate (Kles and Chang, 2006). In addition to providing energy for the host, SCFAs exhibit anti-inflammatory effects via binding to G-protein-coupled receptor 43 (GPR43) (Maslowski et al., 2009), which is expressed in immune cells, including macrophages (Le Poul et al., 2003; Sivaprakasam et al., 2016). Butyrate is the most important SCFA due to its role in antagonizing colonic inflammation (Hamer et al., 2008) and the ability to meet 6–10% of the daily human energy requirements (Bergman, 1990). *Ruminococcaceae*, *Eubacterium*, *Clostridia*, and *Firmicutes* were identified as the main producers of butyrate (Ohira et al., 2017). Moreover, butyrate can exert an anti-inflammatory effect in part by suppressing the activation of NF-κB (Luhrs et al., 2002), a transcription factor that regulates the inflammatory and innate immune responses (Karin et al., 2002). In addition, butyrate strongly inhibits the interferon-gamma (IFN-γ) signaling to ameliorate inflammation (Klampfer et al., 2003). Butyrate also targets peroxisome proliferator-activated receptor-γ (PPARγ) to prevent colon inflammation (Kinoshita et al., 2002).

**TABLE 1:** Factors that influence composition of gut microbiota.

Factor	Classification	Research object	Effects on gut microbiota	References
Host genetics		Twin pairs	Heritable microbes: *Christensenellaceae Methanobrevibacter smithii* (MZ > DZ)	Hansen et al., 2011; Goodrich et al., 2014; Goodrich et al., 2016
	Genetically obese	Mice	↑ *Firmicutes* ↓ *Bacteroidetes*	Turnbaugh et al., 2006
Diet	High-fiber diet	Children from Europe and rural Africa.	↑ *Bacteroidetes* (*Prevotella*) ↓ *Firmicutes*	De Filippo et al., 2010
	High-beef diet	Ten human volunteers	↓ *Bifidobacterium* ↑ *Bacteroides* ↑ *Clostridia*	Hentges et al., 1977
	High-fat diet	Human	↑ *Bacteroides*	Wu et al., 2011
		Rats	↓ *Lactobacillus*	Lecomte et al., 2015
	High-sugar diet	Natural sugars-fed mice (glucose, fructose)	↓ *Bacteroidetes* ↑ *Proteobacteria*	Do et al., 2018
		Artificial sweeteners-fed mice	↑ *Bacteroides* ↓ *Lactobacillus*	Suez et al., 2014
Antibiotics	Macrolide	2–7-year-old Finnish children	↓ *Actinobacteria* ↑ *Bacteroidetes* ↓ *Proteobacteria*	Korpela et al., 2016
	clindamycin	18–45 years healthy volunteers	↓ *lactobacilli* ↓ *bifidobacteria*	Rashid et al., 2015
	Vancomycin	Obese males with metabolic syndrome	↓ gram-positive bacteria (mainly *Firmicutes*) ↑ gram-negative bacteria (mainly *Proteobacteria*)	Vrieze et al., 2014
Inflammation	RA	Human	↑ *Lactobacillus* ↑ *Prevotella copri* ↓ *Bifidobacteria* ↓ *Bacteroides*	Liu et al., 2013; Scher et al., 2013
	IBD	Human	↓ *Firmicutes* ↓ *Bacteroidetes* ↑ *Proteobacteria* ↑ *Adherent-invasive Escherichia coli* (AIEC)	Darfeuille-Michaud et al., 2004; Frank et al., 2007; Fava and Danese, 2011; Matsuoka and Kanai, 2015
	TRUC	Mice	↓ *Firmicutes* (*Clostridium*) ↑ *Bacteroidales* ↑ *Proteus mirabilis* ↑ *Klebsiella pneumoniae*	Garrett et al., 2010

*RA, Rheumatoid arthritis; IBD, inflammatory bowel disease; TRUC, T-bet-/- RAG2-/- ulcerative colitis.*

## Macrophages and Inflammation

Macrophages have been considered to be the central effector cells in many chronic inflammatory diseases (Moore and Tabas, 2011). Macrophages have high functional plasticity (Stout and Suttles, 2004) and exhibit pro- or anti-inflammatory properties in response to various cytokines and microbial products (Mantovani et al., 2004). In the early 1990s, scientists found that interleukin (IL)-4 exerts different effects on macrophage phenotypes compared to IFN-γ and/or LPS, and the concept of alternatively activated macrophages has been proposed (Stein et al., 1992). Since then, macrophages have been divided into two groups: classically activated M1 phenotypes, which are stimulated by IFN-γ and LPS and exert pro-inflammatory effects, and alternatively activated M2 phenotypes, which are stimulated by the IL-4 or IL-13 and perform anti-inflammatory functions, as reviewed by Tang et al. (2019).

Many studies have been focused on applying the macrophage phenotype changes to the treatment of inflammatory diseases. For instance, atherosclerosis is a chronic inflammatory disease in which macrophages play a major role at all stages (Tabas and Bornfeldt, 2016). Ouimet et al. (2015) reported that miR-33 antagonizes atherosclerosis partly through promoting the M2 macrophage polarization. In addition, macrophages are associated with obesity-associated inflammatory diseases of the adipose tissue (Weisberg et al., 2003). A study by Lumeng et al. (2007) illustrated that adipose tissue macrophages (ATMs) switch from the M1 polarized macrophage to the M2 polarized macrophage, thereby reducing the adipose tissue-derived inflammatory signals (Lumeng et al., 2007). Additionally, the RA was found to be closely related to the imbalance of the M1 and M2 macrophages and could be attenuated by reestablishing the macrophage equilibrium (Li et al., 2012). Identically, IBD could be ameliorated via the M1 to M2 switch in the colitis mouse model (Zhu et al., 2016). These findings suggest a strong correlation between macrophages and the inflammatory diseases and imply that macrophages participate in the inflammatory process mainly by shifting from the pro-inflammatory (M1) to anti-inflammatory (M2) phenotype (Porcheray et al., 2005).

## Macrophages Are Involved in the Interaction Between the Gut Microbiota and Obesity

Obesity is a state of low-grade chronic inflammation, characterized by the expanded adipose tissue (AT) in which macrophages account for 1–30% of its composition (Hauner, 2005). As reviewed by Russo and Lumeng (2018), adipose tissue macrophages (ATMs) include tissue-resident and monocyte-derived macrophages, and the increased number of ATMs in AT is mainly due to the recruitment of blood-macrophages and proliferation of resident macrophages. ATMs are the major effectors of the adipose tissue inflammation; ATMs accumulate in AT to participate in the inflammatory pathways, such as increasing the adipose tissue production of pro-inflammatory cytokines (Weisberg et al., 2003). Since scientists have found that the composition of the gut microbiota is significantly different between the lean mice and the obese mice, the latter showing lower *Bacteroidetes* and higher *Firmicutes* bacteria (Ley et al., 2005) levels, people began to realize the close connection between the gut microbiota and obesity. Subsequently, the gut microbiota has been found not only responsible for the weight gain and energy harvest (Samuel et al., 2008), but also involved in the development of the low-grade inflammation associated with obesity (Cani and Delzenne, 2009).

**FIGURE 1:**
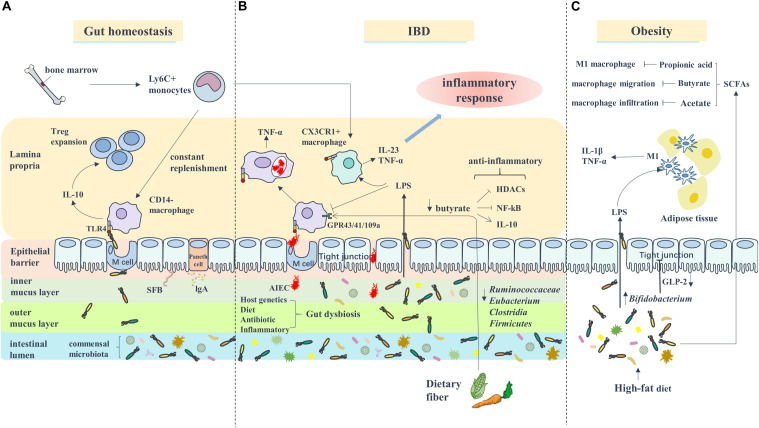
Macrophages are involved in the interaction between gut microbiota and IBD or Obesity. **(A)** Under gut homeostasis, bone marrow derived Ly6C^+^ monocytes are constantly recruited to gut to replenish the intestinal macrophages (CD14^–^ macrophages) which recognize the pathogen through TLR4 receptor and secret anti-inflammatory cytokine IL-10 to promote Treg cell expansion. **(B)** In IBD, the inflammatory environment lead to gut dysbiosis included increased number of AIEC which can survive and replicate in macrophages, and decreased butyrate bacteria like *Ruminococcaceae*, *Eubacterium*, *Clostridia*, and *Firmicutes*, which impairs the ability of butyrate exert anti-inflammatory role through inhibiting HDACs/NF-κB (GPR43/41) or promoting IL-10 secretion (GPR109a). Additionally, blood Ly6C^+^ monocytes are recruited to intestinal to become inflammatory macrophages (CX3CR1^+^ macrophages) and secrete pro-inflammatory cytokines such as IL-23 and TNF-α to participated in inflammatory response. **(C)** In Obesity, the alteration of gut microbiota composition caused by HFD-diet lead to an increase of intestinal permeability, therefore LPS enter system circulation (i.e., metabolic endotoxemia). Adipose tissue macrophages are response to LPS activation and transform to M1 phenotype.

Accumulating evidence indicates that alterations in the gut microbiota are responsible for the progression of obesity. There are two possible pathways of the gut microbiota affecting obesity and the obesity-related diseases, such as diabetes and cardiovascular diseases: the circulation of bacterial components (e.g., LPS) and metabolites SCFAs (Harris et al., 2012) (Figure 1). The obesity-associated decrease in the *Bifidobacterium* levels leads to the reduced production of GLP-2, a key molecule that promotes the intestinal barrier function, eventually destroying the tight junction integrity of the epithelial barrier and enhancing the intestinal permeability (Cani et al., 2009). Under these circumstances, LPS enters circulation through passive diffusion across the intestinal mucosa (Harris et al., 2012). In addition, Caesar et al. (2012) found that the conventionally raised (CONV-R) mice showed increased plasma LPS concentrations compared with the germ-free (GF) mice, both mildly obese. Moreover, gut-derived LPS promoted macrophage accumulation in the adipose tissue (Caesar et al., 2012) and increased the proliferation of the ATMs via a CD14-dependent pathway (Luche et al., 2013). In AT, macrophages recognized LPS through the TLR4 receptor, which is expressed on the cell surface, thereby converting from the M2 phenotype to the M1 phenotype and subsequently secreting pro-inflammatory cytokines, such as IL-1β and TNF-α, to participate in the inflammatory response (Weisberg et al., 2003; Park et al., 2009; Harford et al., 2011). Similarly, some reports reveal that the lack of TLR4 attenuates the adipose tissue inflammation by shifting the ATM polarization toward the M2 phenotype (Orr et al., 2012). In addition, the level of SCFAs was found to be higher in the obese compared with the normal-weight children due to the differences in the gut microbiota (Rahat-Rozenbloom et al., 2014; Riva et al., 2017). Conversely, the HFD-fed mice displayed decreased SCFAs levels (Lu et al., 2016). Abundant reports indicate that macrophages are involved in the SCFA-mediated anti-inflammatory effects in obesity. For example, propionic acid, a major SCFA, prevents the obesity-related inflammation partially through acting on macrophages (Al-Lahham et al., 2012). *In vitro* studies have demonstrated that butyrate could inhibit the LPS-mediated macrophage migration via the suppression of Src (Maa et al., 2010). Also, the circulating acetate level was increased by the antibiotic use in HFD-fed mice, reducing macrophage infiltration via activating AMPK (Carvalho et al., 2012).

## Macrophages Are Involved in the Interaction Between the Gut Microbiota and IBD

Inflammatory bowel disease, which includes UC and CD, is a persistent inflammatory disorder of the intestine. It is becoming increasingly appreciated that the gut dysbiosis is linked to the pathogenesis of IBD (Ni et al., 2017). Under normal conditions, the mucosal immune system is precisely controlled to protect the intestines from the pathogenic bacteria infections and simultaneously maintain the immunological tolerance to benefit the gut microbiota, as described in the accompanying review (Agace and McCoy, 2017). The disruption of normal mucosal immunity toward the commensal microbiota leads to continuous microbial antigenic stimulation and contributes to chronic intestine inflammation (Strober et al., 2007). In IBD, the numbers of the commensal bacteria (predominantly *Firmicutes* and *Bacteroidetes*) were reduced (Frank et al., 2007), and the numbers of the facultative anaerobic bacteria of the family *Enterobacteriaceae* were increased (Zuo and Ng, 2018). Furthermore, the levels of the microbiota metabolites, the SCFAs, decreased along with the reduction in the population of *Clostridium IXa* and *IV* groups, the main butyrate-producing bacteria (Fava and Danese, 2011), limiting intestinal inflammation by regulating the function of colonic T cells (Tregs) (Smith et al., 2013). In a word, the inflammation-related disruption of the host-microbe interactions and decrease in the SCFA levels are involved in the development of IBD (Figure 1).

Intestinal macrophages, which reside in the lamina propria, are among the most abundant immune cells in the gut (Lee et al., 1985) and the first line of defense against the invasion of foreign pathogens (Smythies et al., 2005). Unlike blood monocytes or other tissue-resident macrophages, intestinal macrophages do not respond to LPS due to the absence of CD14, a surface receptor that plays a key role in the LPS-induced cell activation; hence, the LPS-induced pro-inflammatory cytokine production (IL-1, IL-6, IL-8, and TNF-α) is markedly reduced (Ulevitch and Tobias, 1995; Smith et al., 2001; Smythies et al., 2005). Moreover, intestinal macrophages produce anti-inflammatory cytokines like IL-10 and further regulate T-cell differentiation to prevent mucosal auto-inflammation (Denning et al., 2007). Although human intestinal macrophages exhibit profound “inflammatory anergy,” they retain strong phagocytic activity and perform defense functions (Smythies et al., 2005). Since intestinal macrophages perform such essential functions, they are integral to maintaining intestinal homeostasis. Kamada et al. (2008) identified a unique CD-specific macrophage subset that expresses high levels of CD14 and produces pro-inflammatory cytokines, such as IL-23 and TNF-α, leading to the accumulation of pro-inflammatory macrophages. In other words, there are “resident” and “inflammatory” macrophages in the gut that share the same Ly6Chi monocyte precursors under different conditions (Bain et al., 2013).

Although there is a current consensus that gut dysbiosis is closely linked to IBD, it should not be concluded that there is a direct causal relationship between them (Ni et al., 2017). However, it is clear that intestinal inflammation can alter the composition of the gut microbiota, which further exacerbates inflammation (Pickard et al., 2017). Macrophages are well established as the innate immune system cells that recognize invading microbiota using pattern recognition receptors and exhibit efficient phagocytic and bactericidal activity (Janeway and Medzhitov, 2002). Under normal conditions, intestinal macrophages rely on the commensal microbiota for the immune response, while in IBD, inflammatory macrophages are recruited to the inflamed tissue (Grainger et al., 2017). Moreover, the impaired acute inflammatory response in CD leads to the delayed clearance of bacteria (Smith et al., 2009). Adherent-invasive *E. coli* (AIEC), the aggressive functionally altered resident strains in IBD, have the ability to invade intestinal epithelial cells (IEC) (Sartor and Wu, 2017; Palmela et al., 2018). Subsequently, AIEC is swallowed by macrophages and replicates inside them due to the defect in autophagy (Lapaquette et al., 2010), which is involved in the pathogenesis of IBD. Additionally, the SCFA butyrate can exert anti-inflammatory effects via regulating the intestinal macrophage function as a histone deacetylase (HDAC) inhibitor (Chang et al., 2014) or suppressing the NF-κB activation (Luhrs et al., 2002). Furthermore, butyrate has the ability to exhibit anti-inflammatory activity on intestinal macrophages, which express Gpr109a by inducing the expression of IL-10 (Singh et al., 2014), while these functions are attenuated in IBD due to the decrease of butyrate concentration.

## Conclusion

This review provides current understanding of the role of macrophages in gut microbiota and inflammation interactions. On the one hand, the polarization of macrophages, mediated by gut-derived LPS and its metabolites SCFA, plays a key role in the regulation of inflammatory diseases. On the other hand, macrophages are the main players in ensuring intestinal homeostasis through pathogen recognition and elimination and production of anti-inflammatory cytokines. Besides, a recent report by Earley et al. (2018) revealed that a loss of intestinal macrophages influenced the establishment of the gut microbiota in zebrafish, which suggests a critical role of macrophages in shaping the gut microbiota. Taking into account the important role of macrophages in the relationship between gut microbiota and inflammation, developing macrophage-targeting approaches in the prevention and therapy of inflammatory diseases is an appealing strategy. However, the related molecular signaling pathways involved in the roles of macrophages between gut microbiota and inflammation diseases are remained to be defined. Further work is needed to apply macrophages as a perspective target in the treatment of gut microbiota-related inflammatory diseases, like obesity and non-alcoholic fatty liver.

## Author Contributions

JW wrote the manuscript. Y-DW and W-DC initiated the idea for writing, revised the manuscript, and secured the funding for this work.

## Conflict of Interest

The authors declare that the research was conducted in the absence of any commercial or financial relationships that could be construed as a potential conflict of interest.
