# Supplementary material for: The Relationship Between Gut Microbiota and Inflammatory Diseases: The Role of Macrophages
Source: Front Microbiol. 2020 Jun 9;11:1065. doi: 10.3389/fmicb.2020.01065 (PMC7296120; doi:10.3389/fmicb.2020.01065)
Supplement: Supplementary file 1 [file Data_Sheet_1.pdf]

Supplementary Table 1. Role of the gut microbiota in the protection or induction of inflammation diseases.

Microbe	Subject	Disease	The possible role of macrophages	Reference
**Microbiota that promote the development of inflammation disease**				
E. coli	Rats	NASH	Escherichia Coli-LPS induces liver macrophages activation towards pro-inflammation phenotype through the TLR4 pathway	(Zhu et al., 2013; Carpino et al., 2019)
Prevotella copri	Human	RA	The activated macrophages are the main source of pro-inflammatory cytokines and chemokines such as TNF and IL-1β	(Bernard, 2014; Siouti and Andreakos, 2019)
Bacteroides fragilis	NOD mice	T1DM	B. fragilis enhances the phagocytic functions of macrophages and polarize them to an M1 phenotype.	(Deng et al., 2016; Sofi et al., 2019)
Clostridium difficile	Child	Asthma	C. difficile release TcdA and TcdB toxins, which induce the recruitment of macrophages, and survive in the macrophages	(Paredes-Sabja et al., 2012; Salameh et al., 2020)
Adherent-invasive Escherichia coli	Human	CD	Adhere to intestinal epithelial cells, invade epithelial cells, survive and replicate within macrophages.	(Darfeuille-Michaud et al., 2004)
**Microbiota that protect against inflammation diseases**				
Prevotella histicola	HLA-DR3.DQ8 transgenic mice	MS	The induction of suppressive macrophages which produced higher IL-10 and lower IL-12, thus induced a decreased capacity of antigen presentation	(Mangalam et al., 2017)

Roseburia	Human	T2DM	Production of butyrate which exert anti-inflammatory role on macrophages	(Liu et al., 2012; Vrieze et al., 2012)
Lactobacillus helveticus	Mice	Asthma	L. gasseri decreased the numbers of alveolar macrophages which produce IL-17	(Jan et al., 2012; Hsieh et al., 2018)

NASH, nonalcoholic steatohepatitis; MS, Multiple sclerosis; RA, rheumatoid arthritis; T2DM, type 2 diabetes mellitus

References

Bernard, N.J. (2014). Rheumatoid arthritis: Prevotella copri associated with new-onset untreated RA. *Nat Rev Rheumatol* 10(1), 2. doi: 10.1038/nrrheum.2013.187.

Carpino, G., Del Ben, M., Pastori, D., Carnevale, R., Baratta, F., Overi, D., et al. (2019). Increased liver localization of lipopolysaccharides in human and experimental non-alcoholic fatty liver disease. *Hepatology*. doi: 10.1002/hep.31056.

Deng, H., Li, Z., Tan, Y., Guo, Z., Liu, Y., Wang, Y., et al. (2016). A novel strain of Bacteroides fragilis enhances phagocytosis and polarises M1 macrophages. *Sci Rep* 6, 29401. doi: 10.1038/srep29401.

Hsieh, M.H., Jan, R.L., Wu, L.S., Chen, P.C., Kao, H.F., Kuo, W.S., et al. (2018). Lactobacillus gasseri attenuates allergic airway inflammation through PPARgamma activation in dendritic cells. *J Mol Med (Berl)* 96(1), 39-51. doi: 10.1007/s00109-017-1598-1.

Jan, R.L., Yeh, K.C., Hsieh, M.H., Lin, Y.L., Kao, H.F., Li, P.H., et al. (2012). Lactobacillus gasseri suppresses Th17 pro-inflammatory response and attenuates allergen-induced airway inflammation in a mouse model of allergic asthma. *Br J Nutr* 108(1), 130-139. doi: 10.1017/s0007114511005265.

Liu, T., Li, J., Liu, Y., Xiao, N., Suo, H., Xie, K., et al. (2012). Short-chain fatty acids suppress lipopolysaccharide-induced production of nitric oxide and proinflammatory cytokines through inhibition of NF-kappaB pathway in RAW264.7 cells. *Inflammation* 35(5), 1676-1684. doi: 10.1007/s10753-012-9484-z.

Mangalam, A., Shahi, S.K., Luckey, D., Karau, M., Marietta, E., Luo, N., et al. (2017). Human Gut-Derived Commensal Bacteria Suppress CNS Inflammatory and Demyelinating Disease. *Cell Rep* 20(6), 1269-1277. doi: 10.1016/j.celrep.2017.07.031.

Paredes-Sabja, D., Cofre-Araneda, G., Brito-Silva, C., Pizarro-Guajardo, M., and Sarker, M.R. (2012). Clostridium difficile spore-macrophage interactions: spore survival. *PLoS One* 7(8), e43635. doi: 10.1371/journal.pone.0043635.

Salameh, M., Burney, Z., Mhaimeed, N., Laswi, I., Yousri, N.A., Bendriss, G., et al. (2020). The role of gut microbiota in atopic asthma and allergy, implications in the understanding of disease pathogenesis. *Scand J Immunol* 91(3), e12855. doi: 10.1111/sji.12855.

Siouti, E., and Andreakos, E. (2019). The many facets of macrophages in rheumatoid arthritis. *Biochem Pharmacol* 165, 152-169. doi: 10.1016/j.bcp.2019.03.029.

Sofi, M.H., Johnson, B.M., Gudi, R.R., Jolly, A., Gaudreau, M.C., and Vasu, C. (2019). Polysaccharide A-Dependent Opposing Effects of Mucosal and Systemic Exposures to Human Gut Commensal Bacteroides fragilis in Type 1 Diabetes. *Diabetes* 68(10), 1975-1989. doi: 10.2337/db19-0211.

Vrieze, A., Van Nood, E., Holleman, F., Salojärvi, J., Kootte, R.S., Bartelsman, J.F., et al. (2012). Transfer of intestinal microbiota from lean donors increases insulin sensitivity in individuals with metabolic syndrome. *Gastroenterology* 143(4), 913-916.e917. doi: 10.1053/j.gastro.2012.06.031.

Zhu, L., Baker, S.S., Gill, C., Liu, W., Alkhouri, R., Baker, R.D., et al. (2013). Characterization of gut microbiomes in nonalcoholic steatohepatitis (NASH) patients: a connection between endogenous alcohol and NASH. *Hepatology* 57(2), 601-609. doi: 10.1002/hep.26093.
